# Supplementary material for: Mammalian brain glycoproteins exhibit diminished glycan complexity compared to other tissues
Source: Nat Commun. 2022 Jan 12;13:275. doi: 10.1038/s41467-021-27781-9 (PMC8755730; doi:10.1038/s41467-021-27781-9)
Supplement: Supplementary file 1 — Supplementary Information [file 41467_2021_27781_MOESM1_ESM.pdf]

Mammalian brain glycoproteins exhibit diminished glycan complexity compared to other tissues

Sarah E. Williams, Maxence Noel, Sylvain Lehoux, Murat Cetinbas, Ramnik J. Xavier, Ruslan Sadreyev, Edward M. Scolnick, Jordan W. Smoller, Richard D. Cummings, and Robert G. Mealer

Supplementary Information

Supplementary Notes	2
Supplementary Figure 1. MS/MS confirmation of brain N-glycans	5
Supplementary Figure 2. Predicted LacdiNAc structures are uncommon on N-glycans, and O-glycan fucose and NeuAc modification appear in competition	6
Supplementary Figure 3. MS/MS confirmation of brain O-glycans	7
Supplementary Figure 4: Optimization of brain lectin blots	8
Supplementary Figure 5: Glycosidase sensitivity confirms the unique enrichment of high mannose/hybrid N-glycans in brain	9
Supplementary Figure 6: FUMA analysis revealed similar pattern of glycosylation gene regulation across brain regions	10
Supplementary Table 1. Brain protein O-glycan structure, name, mass, and characteristics	11
Supplementary Table 2. Plasma protein N-glycan structure, name, mass, and characteristics	12
Supplementary Table 3. Sex differences in classes of N-glycans	13

Supplementary Notes

Supplementary Note 1. Definitions of glycan classes and structural characteristics

Pauci-mannose - structures including Man-2, Man-3, and Man-4 with no terminal branching or modifications aside from core fucose.

High-mannose - structures including Man-5, Man-6, Man-7, Man-8, and Man-9 with no terminal branching of modifications aside from core fucose.

Hybrid - structures containing GlcNAc extension from the α-1,3 core Man (one or two GlcNAc), and Man extension from the α-1,6 core Man.

Bisected - structures containing GlcNAc extension directly from the core Man residue attached to the chitobiose core, in addition to both α-1,3 Man and α-1,6 Man.

Antenna - refers to the number of GlcNAc attachments to the α-1,3 Man and α-1,6 Man residues of the core structure, and does not include bisected GlcNAc.

Supplementary Note 2. MS/MS confirms common structural components of N-glycans

Glycan structures are incredibly diverse, and in some instances, different arrangements of similar monosaccharide components have the same *m/z,* and thus cannot be distinguished by standard mass spectrometry. Therefore, additional analyses are necessary to confirm the structures of glycans detected using MALDI-MS. Tandem mass spectrometry (MS/MS) allows for a single *m/z* peak to be split into fragment ions, which are analyzed to determine the structure (or structures) of the parent peak in question. We performed MS/MS on several prominent N-glycans from the cortex and cerebellum and obtained complementary results from both regions. We initially presumed several MALDI-MS peaks corresponded to complex, non-bisected N-glycans found in plasma. However, after additional experiments described below, we determined that these peaks primarily represent bisected and/or hybrid N-glycans, further distinguishing the brain N-glycome from that of other tissues.

MS/MS analysis of the most abundant N-glycan, Man-5 (*m/z*: 1579), showed fragment ions consistent with the predicted high-mannose parent structure, including fragments lacking a single mannose (*m/z:* 1361) or a single GlcNAc (*m/z*: 1302), as well as a free hexose (*m/z*: 260) and core GlcNAc (*m/z:* 282, 300) (**Supplementary Figure 1A**). The peak at *m/z*: 1835.9 (~10% abundance in most brain regions) could represent several potential structures including a biantennary glycan with two terminal GlcNAc residues and a core fucose (FA2) commonly found in plasma, a bisected glycan with one antennary GlcNAc (FA1B), or the so-called “LacdiNAc” motif terminating in GalNAc-β1,4-GlcNAc (**Supplementary Figure 1B)**. MS/MS results showed a bisecting GlcNAc residue and a terminal mannose, indicating the presence of FA1B in the parent peak. Several fragment ions from MS/MS results rule in a bisected structure, including a free hexose (*m/z:* 260), which indicates the presence of terminal mannose, and the corresponding structure lacking a terminal mannose (*m/z:* 1617). Additionally, the fragments at *m/z:* 671 and 949 can only result from the fragmentation of a glycan with a bisecting GlcNAc residue. Glycomic analysis of a mouse lacking *Mgat3*, which encodes the only enzyme capable of creating bisected N-glycans (GnT-III), showed a dramatic loss of this glycan in the mutant mouse brain compared to wild-type, providing additional support that *m/z*: 1835.9 represents a bisected species[1]. The presence of the LacdiNAc structure was not supported by our MS/MS results of 1835.9, as the diagnostic fragments at *m/z:* 527 and 545 were not detected

(**Supplementary Figure 2B**). Finally, a potentially sialylated version of a LacdiNAc N-glycan with unambiguous predicted mass of *m/z:* 2442.2 was not present in our samples (**Supplementary Figure 2A**), suggesting that if the LacdiNAc structure is present in the brain, its relative abundance is low.

Additional MS/MS analyses revealed that some peaks consist of a mixture of at least two unique glycans. For example, MS/MS analysis of *m/z*: 2214 generated fragment ions that indicate the presence of both a bisected and non-bisected structure (F2A1G1B, F2A2G1) (**Supplementary Figure 1C**). The major peaks *m/z*: 1576 and 1955 could be formed from either parent structure, but the fragment ions at *m/z*: 195 and 486 are specific for F2A2G1, while *m/z:* 671 and 1154 are specific for F2A1G1B. MS/MS analysis of the peak at *m/z*: 2244 contained a mixture of bisected hybrid glycans, with either a Lewis X (Le^X) epitope and four mannose residues (A1FG1BH4) or a core fucose and five mannose residues (FA1BH5) (**Supplementary Figure 2D**). Fragment ions consistent with A1FG1BH4 include the Le^X fragment (*m/z*: 660) and the corresponding glycan missing Le^X (*m/z*: 1606). Fragment ions consistent with FA1BH5 include fragments missing both the core GlcNAc and core fucose (*m/z:* 1792) as well as several smaller fragments resulting from the tri-mannose hybrid arm (*m/z:* 196, 431 and 450). In sum, our N-glycan MS/MS results are consistent with the predominance of high-mannose, bisected, and fucosylated structures, lower galactose-containing structures, and a very small amount of sialylated N-glycans in the mouse brain, and emphasize the importance of confirming glycan structures with several techniques before proceeding with additional analyses.

Supplementary Note 3. Limitations of O-glycan purification and analysis.

We initiated our studies with planned groups of at least 6 wild-type mice per sex across 4 glycans for both N- and O-glycans. Several lines of evidence suggest that O-glycans are far less abundant in the brain relative to N-glycans, including the lectin blots presented in Fig. 4, and the results of our quantitative studies of sialic acid levels in the brain[2]. The O-glycan purification protocol requires several additional steps beyond dialysis of lipids, trypsinization, and removal of N-glycans by PNGase F, including glycopeptide elution, *β*-elimination, purification, permethylation, and isolation for MS analysis. We have observed that some brain O-glycan samples do not produce any detectable signal by MALDI-MS, presumably as these low abundance structures are lost somewhere during the purification and isolation due to a technical issue (Fig. 5D). We aimed to have at least 3 samples/group for comparisons (with a mean +/- SEM), though some groups, for example female cortex O-glycans, only yielded 2 analyzable samples. We have chosen to report the data from these underpowered groups while not performing any statistical comparison or drawing premature conclusions in hopes that the data can be useful for qualitative rather than quantitative comparisons. In addition, a complementary study with larger sample sizes for both brain regions and sex of mice harboring a single point mutation produced identical cortex O-glycan profiles compared to the wild-type mice reported here[2]. As such, we have observed a consistent O-glycan pattern across multiple samples but refrain from drawing concrete conclusions within this group.

Supplementary Note 4. MS/MS confirms the presence of both O-GalNAc and O-mannose glycans in the brain

Several of the peaks detected from brain O-glycan samples could correspond to either an O-GalNAc or an O-mannose glycan, so we performed MS/MS of highly abundant O-glycans to confirm their structural composition. The most abundant O-glycan peak across all brain regions was *m/z:* 1256, predicted to represent a core 1 O-GalNAc structure modified by two NeuAc residues. We confirmed the structural composition of this glycan, identifying fragments containing NeuAc bound to both the Gal (*m/z:* 620) and GalNAc residues (*m/z:* 659)

(**Supplementary Figure 2A**). The peak at *m/z:* 895 also contains a core 1 O-GalNAc glycan, modified by one NeuAc residue, and MS/MS results indicated two structural conformations present (**Supplementary Figure 2B**). The fragment ions at *m/z:* 620, 316, 298, 245, and 227 all indicate the presence of a core 1 O-glycan with NeuAc attached to the Gal residue. Additionally, the fragments at *m/z:* 677, 659, 284, and 259 correspond to a parent glycan with the sialic acid attached to the core GalNAc residue.

The second most abundant O-glycan in the brain, at *m/z:* 1099, could correspond to three possible parent glycans: an O-GalNAc structure modified with one NeuAc and two Gal residues, an O-GalNAc structure with one Gal, one Fuc, and one NeuGc residue, or an O-mannose glycan extended by GlcNAc, Gal and NeuAc. Fragmentation of *m/z:* 1099 revealed fragment ions exclusive to the O-mannose structure, including core Man (*m/z:* 275) and the corresponding trisaccharide from this cleavage (*m/z:* 847) (**Supplementary Figure 2C**). Glycomic analysis of a mouse lacking *Pomgnt1*, which encodes the enzyme necessary for extension of O-mannose glycans, revealed a complete loss of *m/z:* 1099 in the mutant mouse brain, supporting the classification of this peak as an O-mannose structure[3].

The peak at *m/z:* 912 was also absent in the *Pomgnt1* knock-out mouse brain, providing evidence that it contains an O-mannose type glycan with a Le^X extension, as opposed to a fucosylated O-GalNAc glycan with the same mass. Our MS/MS results confirmed the structure of the O-mannose glycan at *m/z*: 912, with the identification of fragment ions such as the core Man and Le^X epitope (*m/z:* 275, 660) (**Supplementary Figure 2D**). Of note, the *Pomgnt1* knock-out mouse brain retained O-GalNAc glycans, including *m/z:* 1256 and 895, corroborating our determination of these glycans as O-GalNAc-type.

Supplementary References:

1. Nakano, M. *et al.* Bisecting GlcNAc Is a General Suppressor of Terminal Modification of N-glycans. *Mol Cell Proteomics* **18**, 2044–2057 (2019).
2. Mealer, R. G. *et al. The schizophrenia-associated variant in* SLC39A8 *alters N-glycosylation in the mouse brain*. http://biorxiv.org/lookup/doi/10.1101/2020.12.22.424076 (2020) doi:10.1101/2020.12.22.424076.
3. Stalnaker, S. H. *et al.* Glycomic Analyses of Mouse Models of Congenital Muscular Dystrophy. *J. Biol. Chem.* **286**, 21180–21190 (2011).

Supplementary Figures

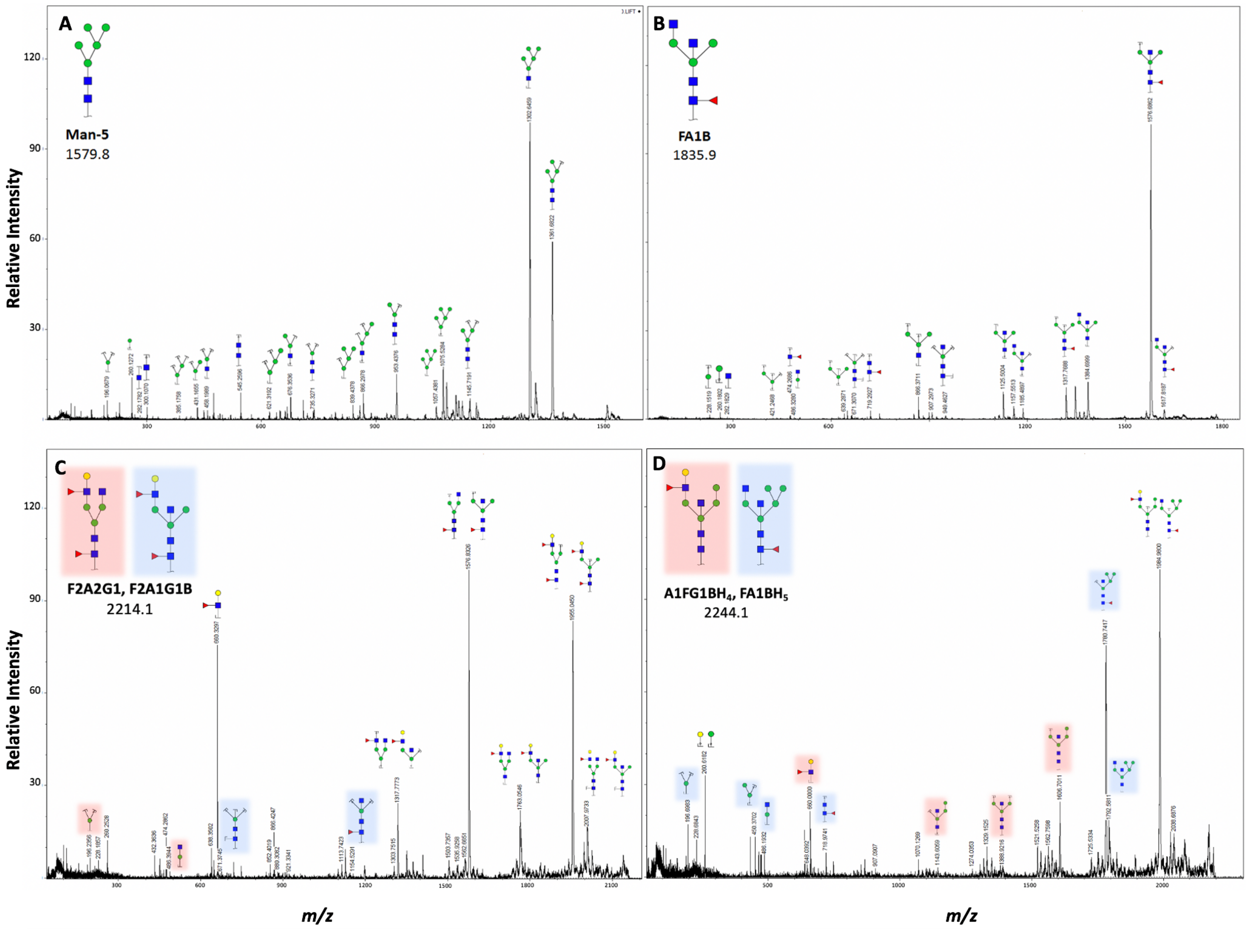

Supplementary Figure 1. MS/MS confirmation of mouse brain N-glycans. A) MS/MS analysis of m/z: 1579 generated fragment ions all consistent with Man-5. B) Fragmentation of m/z: 1836 revealed the presence of a bisected glycan with a terminal mannose. C) Fragmentation of m/z: 2214 and D) m/z: 2244 both indicated two distinct glycan structures at each peak, ruling in that both distinct isomeric structures (with the same m/z mass on MS) exist within the sample.

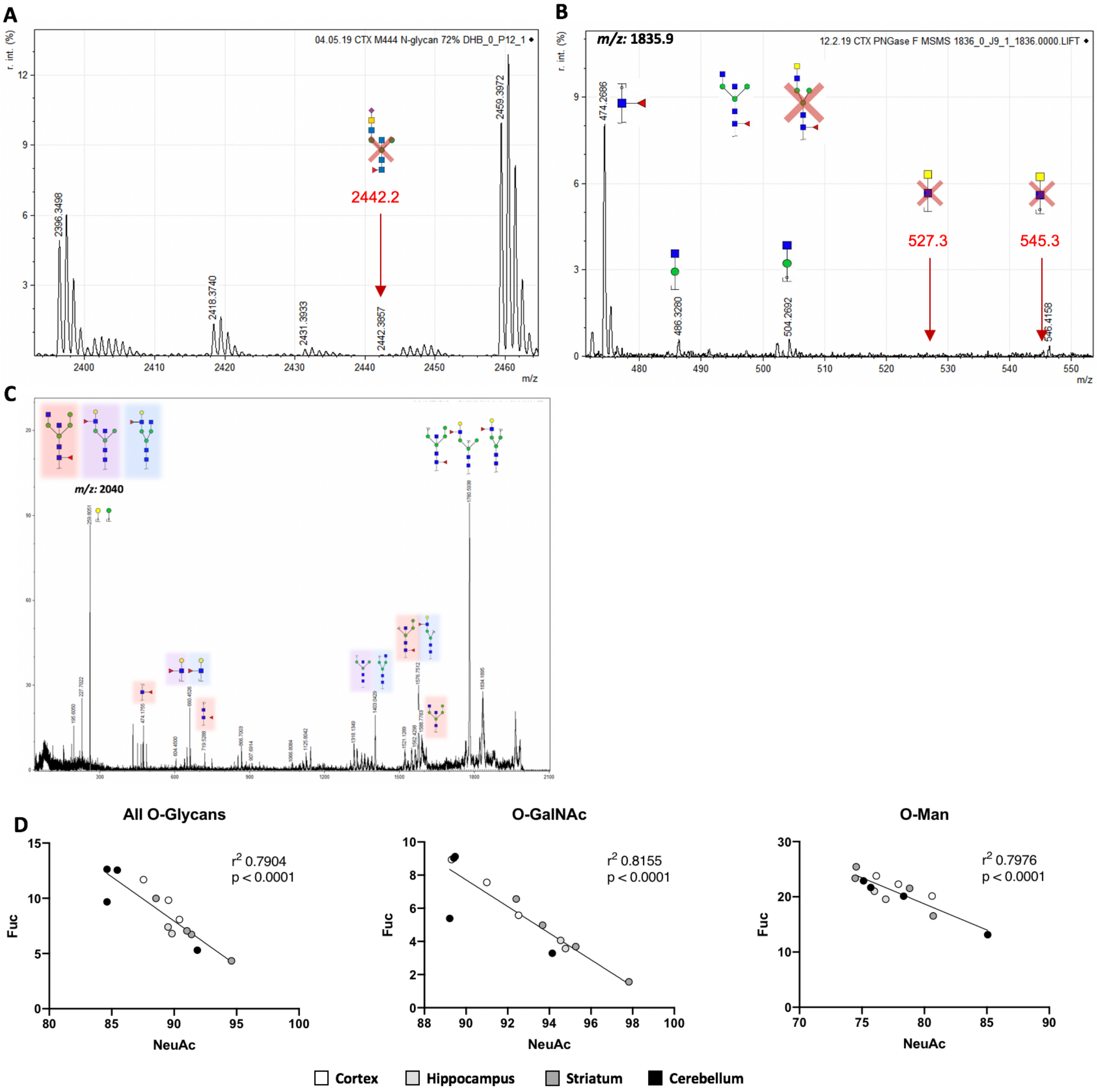

Supplementary Figure 2. Predicted LacdiNAc structures are uncommon on N-glycans, and O-glycan fucose and NeuAc modification appear in competition. A) MS spectrum of cortex N-glycans do not have a peak correlating with the unambiguous LacdiNAc structure at m/z: 2442 (abundance < 0.05%, S/N ratio 1.4). B) MS/MS analysis of the bisected N-glycan peak at m/z 1836, as confirmed in Supplementary Figure 1B, which could also represent an unsialylated LacdiNAc structure, does not reveal any diagnostic fragments to rule in this structure, namely the LacdiNAc fragments at either m/z: 527.3 or 545.3. C) MS/MS analyses of m/z: 2040 revealed a mixture of distinct glycan structures contribute to this single isomeric mass on MS, but none diagnostic for LacdiNAc structures. D) Simple linear regression analysis demonstrated that the abundance of NeuAc-containing O-glycans is negatively correlated with the amount of fucose-containing O-glycans within a sample. This trend is observed in total O-glycans, O-GalNAc-type, and O-mannose-type glycans. Each data point represents a brain region from each mouse, color coded by region, with r^2 and *p*-values indicated for each regression analysis.

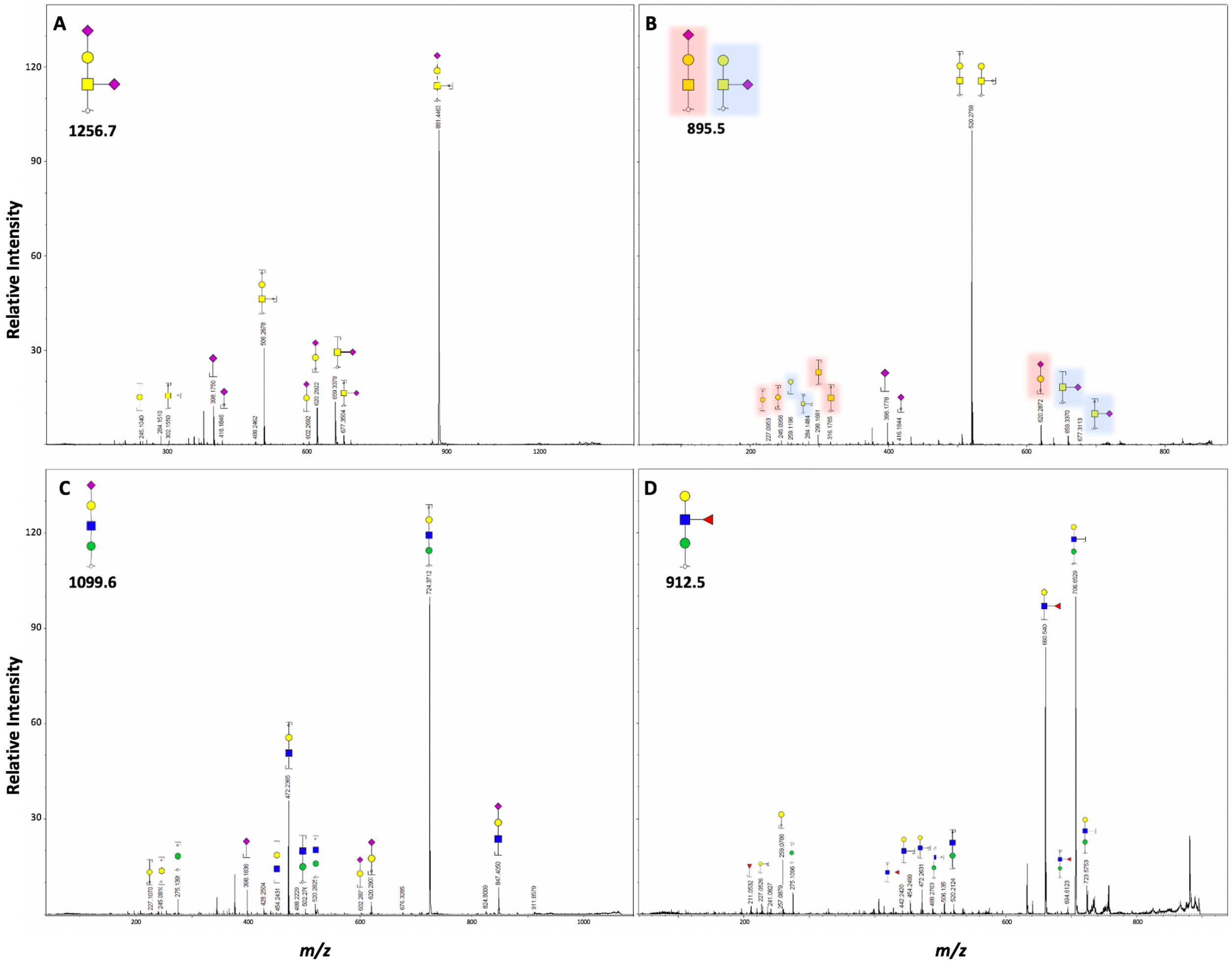

Supplementary Figure 3. MS/MS confirmation of mouse brain O-glycans. A) MS/MS analysis of the most abundant O-glycan peak, m/z: 1256, generated fragment ions consistent with a di-sialylated core-1 O-GalNAc glycan. B) Fragmentation of m/z: 895 identified ions representative of mono-sialylated core-1 O-GalNAc glycans with NeuAc attached to either GalNAc or Gal, confirming that both isomeric structures are present in the sample. C) Fragmentation of m/z: 912 and D) m/z: 1099 confirm the presence of O-mannose glycans with either sialic acid or fucose.

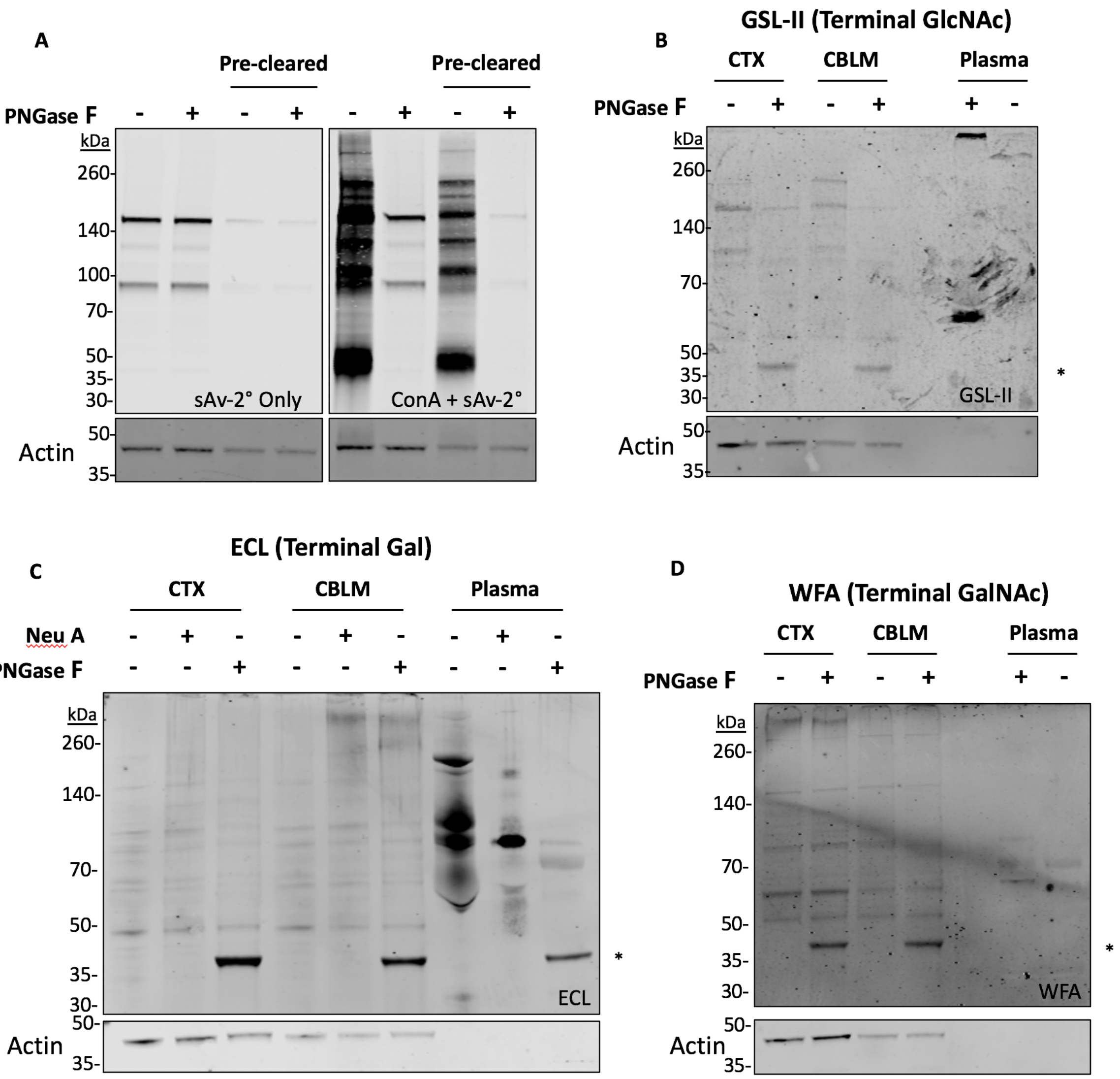

Supplementary Figure 4: Optimization of mouse brain lectin blots. A) Streptavidin bead pre-clearing of brain lysate reduces non-specific binding to biotin-bound proteins. Protein from mouse frontal cortex was incubated with or without magnetic streptavidin beads for 1 hour, followed by PNGase F digestion. 15 µg of protein was loaded in each lane. Left panel was incubated with fluorescent streptavidin secondary alone (LiCOR sAv-800λ); right panel was incubated with primary biotinylated ConA followed by fluorescently labeled streptavidin secondary (LiCOR sAv-800λ). Protein lysate from mouse cortex and cerebellum with human plasma as a positive control was treated with or without PNGase F and the sialidase Neu A and visualized using biotinylated lectins GSL-II (B), ECL (C), or WFA (D), which recognize terminal GlcNAc, Gal, and GalNAc, respectively. A small amount of PNGase F-dependent binding of GSL-II is noted from brain, as well as a small amount of ECL binding insensitive to PNGase F, consistent with terminal GlcNAc on N-glycans and terminal Gal on O-glycans, and complements our glycomic results. No PNGase F-dependent WFA binding is observed, suggesting that N-glycans with terminal GalNAc, such as that found in the LacdiNAc structure, are uncommon. Non-specific binding of lectins to PNGase F is noted by an asterisk (*) near 35 kDa. Protein blotting of brain lysate with each lectin has been repeated at least three times each with similar results. Source data are provided as a Source Data file.

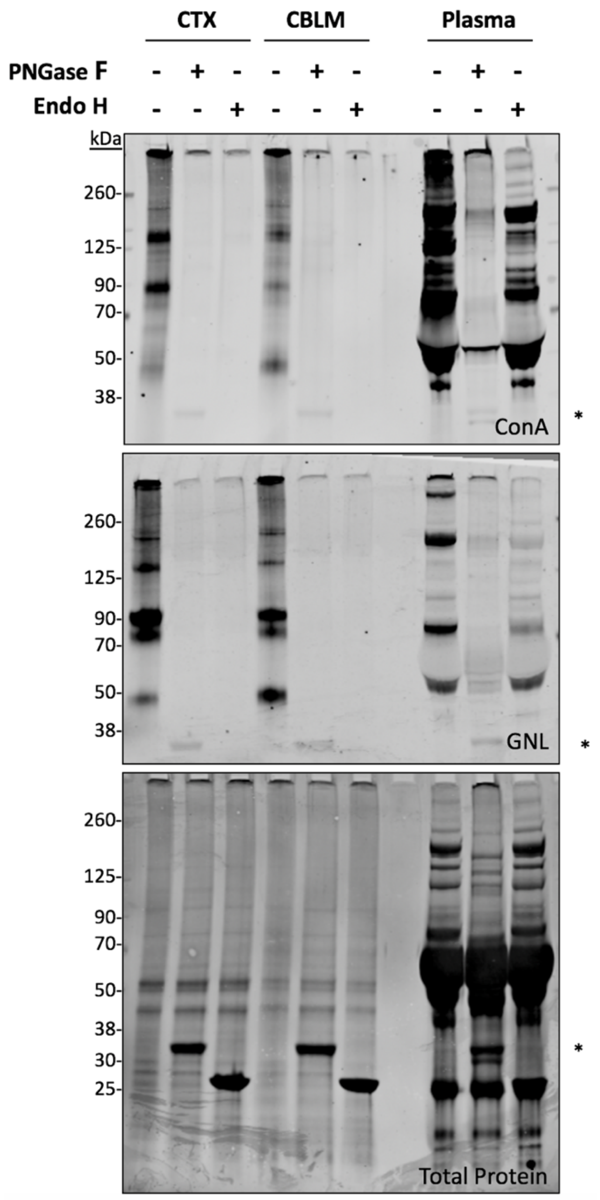

Supplementary Figure 5: Glycosidase sensitivity confirms the unique enrichment of high mannose/hybrid N-glycans in brain. Streptavidin-cleared lysate from mouse cortex (CTX) and cerebellum (CBLM), in addition to human plasma, were analyzed by lectin blotting with ConA and GNL before and after PNGase F and Endo H treatment. 15 µg of brain protein lysate and 50 µg of plasma protein was loaded in each lane, followed by incubation with primary biotinylated lectins and fluorescently labeled streptavidin secondary (LiCOR sAv-800λ) and Total Protein stain. All ConA and GNL staining in CTX and CBLM was removed with either PNGase F (removes all N-glycans) or Endo H (removes only high mannose/hybrid N-glycans), confirming that the dominant N-glycan species in brain are high mannose/hybrid. In contrast, all plasma N-glycans were removed by PNGase F while many were insensitive to Endo H, consistent with a low relative abundance of high mannose/hybrid structures and more complex N-glycans. Non-specific binding of lectins to PNGase F is noted by an asterisk near 35 kDa (*). Protein blotting of brain lysate with each lectin has been repeated at least three times each with similar results Source data are provided as a Source Data file.

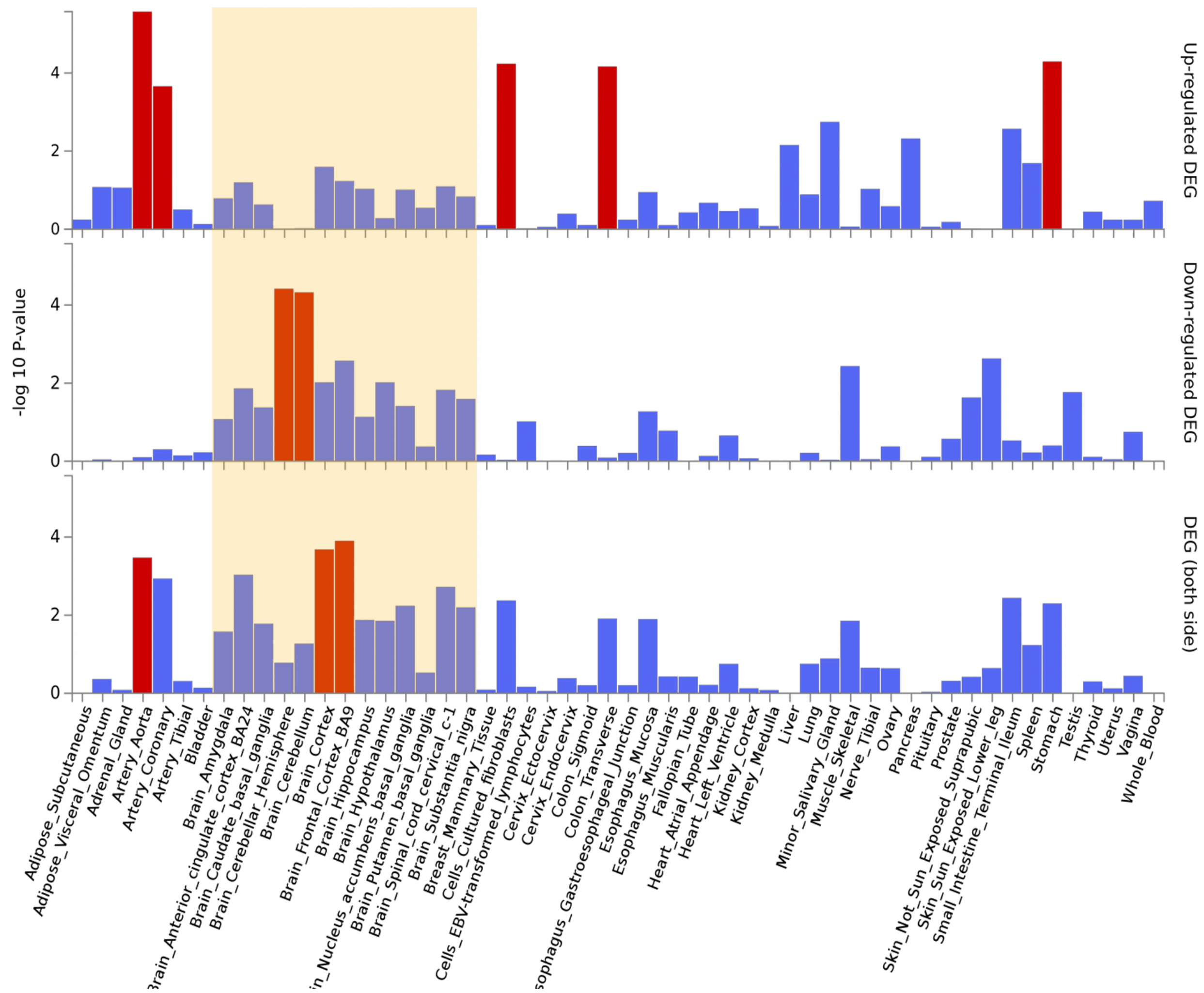

Supplementary Figure 6: FUMA analysis revealed similar pattern of glycosylation gene regulation across brain regions. Human tissue specific analysis shows some regional differences across 13 independent brain regions, but most show an overall pattern of downregulation, with significantly enriched DEG sets ($P_{bon} < 0.05$ and absolute fold change ≥ 0.58) highlighted in red using a two-tailed t-test.

Supplementary Tables

Supplementary Table 1. Brain protein O-glycan structure, name, mass, and characteristics.

Glycan Name	*m/z*	HexNAc	Hexose	NeuAc	NeuGc	All Sia	Fucose	Ser/Thr O-Man	Ser/Thr O-GalNAc	Ambiguous
HexNAcHex	**534.3**	1	1	0	0	0	0	0	0	1
HexNAcNeuAc	**691.4**	1	0	1	0	1	0	0	1	0
HexNAc2Hex	**779.4**	2	1	0	0	0	0	0	1	0
HexNAcHexNeuAc	**895.5**	1	1	1	0	1	0	0	1	0
HexNAcHex2Fuc	**912.5**	1	2	0	0	0	1	1	0	0
HexNAcHexNeuGc	**925.5**	1	1	0	1	1	0	0	1	0
HexNAc2Hex2	**983.5**	2	2	0	0	0	0	0	1	0
HexNAcHexNeuAcFuc	**1069.6**	1	1	1	0	1	1	0	1	0
HexNAcHex2NeuAc	**1099.6**	1	2	1	0	1	0	1	0	0
HexNAcHexNeuGc	**1129.6**	1	2	0	1	1	0	1	0	0
HexNAc2Hex2Fuc	**1157.6**	2	2	0	0	0	1	0	1	0
HexNAcHexNeuAc2	**1256.6**	1	1	2	0	2	0	0	1	0
HexNAcHexNeuAcNeuGc	**1286.7**	1	1	1	1	2	0	0	1	0
HexNAcHexNeuGc2	**1316.7**	1	1	0	2	2	0	0	1	0
HexNAc2Hex2NeuAc	**1344.7**	2	2	1	0	1	0	0	0	1
HexNAc2Hex3Fuc	**1361.7**	2	3	0	0	0	1	1	0	0
HexNAcHex2NeuAc2	**1460.8**	1	2	2	0	2	0	1	0	0
HexNAc2Hex2NeuAcFuc	**1518.8**	2	2	1	0	1	1	0	0	1
HexNAc2Hex3Fuc2	**1535.8**	2	3	0	0	0	2	1	0	0
HexNAc2Hex3NeuAc	**1548.8**	2	3	1	0	1	0	1	0	0
HexNAc2Hex3NeuGc	**1589.8**	2	3	0	1	1	0	1	0	0
HexNAcHexNeuAc3	**1617.8**	1	1	3	0	3	0	0	1	0
HexNAc2Hex2NeuAc2	**1705.9**	2	2	2	0	2	0	0	1	0
HexNAc2Hex3NeuAcFuc	**1722.9**	2	3	1	0	1	1	1	0	0
HexNAc2Hex3NeuAc2	**1910.0**	2	3	2	0	2	0	1	0	0
HexNAcHexNeuAc4	**1979.1**	1	1	4	0	4	0	0	1	0

Supplementary Table 2. Plasma protein N-glycan structure, name, mass, and characteristics.

Glycan Name	*m/z*	GlcNAc	Mannose	Galactose	NeuGc	Fucose	High-Man	Hybrid	Antenna
Man-5	**1579.9**	2	5	0	0	0	1	0	0
A1G1	**1620.9**	3	3	1	0	0	0	0	1
A2	**1661.9**	4	3	0	0	0	0	0	2
Man-6	**1784.0**	2	6	0	0	0	1	0	0
FA2	**1836.0**	4	3	0	0	1	0	0	2
A2B	**1907.1**	5	3	0	0	0	0	0	3
Man-7	**1988.1**	2	7	0	0	0	1	0	0
A1G1S1	**2012.2**	3	3	1	1	0	0	0	1
FA2G1	**2040.2**	4	3	1	0	1	0	0	2
Man-8	**2192.2**	2	8	0	0	0	1	0	0
A1G1S1H	**2216.2**	3	4	1	1	0	0	1	1
A2G1S1	**2257.3**	4	3	1	1	0	0	0	2
Man-9	**2396.3**	2	9	0	0	0	1	0	0
A1G1S1H	**2420.3**	3	5	1	1	0	0	1	1
FA2G1S1	**2431.4**	4	3	1	1	1	0	0	2
A2G2S1	**2461.4**	4	3	2	1	0	0	0	2
FA2G2S1	**2635.5**	4	3	2	1	1	0	0	2
A2G2S2	**2852.6**	4	3	2	2	0	0	0	2
A3G3S1	**2910.6**	5	3	3	1	0	0	0	3
FA2G2A2	**3026.7**	4	3	2	2	1	0	0	2
A2G2S3	**3243.8**	4	3	2	3	0	0	0	2
A3G3S2	**3301.8**	5	3	3	2	0	0	0	3
FA2G2S3	**3417.9**	4	3	2	3	1	0	0	2
A3G3S3	**3693.0**	5	3	3	3	0	0	0	3
A3FG3S3	**3867.1**	5	3	3	3	1	0	0	3
A3G3S4	**4084.3**	5	3	3	4	0	0	0	3
A3FG3S4F	**4258.4**	5	3	3	4	1	0	0	3
A3G3S5	**4475.3**	5	3	3	5	0	0	0	3
A4G4S4	**4533.5**	6	3	4	4	0	0	0	4

Supplementary Table 3. Sex differences in classes of N-glycans.

	Plasma			Cortex			Cerebellum		
**Glycan Category**	**Male**	**Female**	***p-value***	**Male**	**Female**	***p-value***	**Male**	**Female**	***p-value***
Paucimannose	-	-	-	3.10	3.33	0.43	1.17	2.87	0.12
High mannose	1.1	1.1	0.87	62.8	59.8	0.34	61.3	59.9	0.67
Mono-antennary	1.2	0.9	0.47	18.9	20.3	0.24	12.9	16.5	0.09
Bi-antennary	75.4	80.8	**0.046***	13.5	14.2	0.71	21.7	17.2	**0.047***
Tri-antennary	22.2	17.2	0.07	1.5	2.2	0.21	2.6	3.0	0.48
Tetra-antennary	0.1	0.1	0.30	0.09	0.14	0.46	0.3	0.6	0.13
Hybrid	0.8	0.6	0.46	5.2	6.3	0.22	8.3	12.1	0.07
Bisected	-	-	-	30.2	31.9	0.53	34.4	32.0	0.50
Fucose	9.4	47.7	**0.00006*****	34.5	36.9	0.39	35.2	34.3	0.72
Galactose	98.2	98.2	0.99	13.1	14.4	0.52	14.0	14.9	0.65
NeuAc	-	-	-	1.5	2.2	0.42	2.8	4.8	**0.040***
NeuGc	97.7	97.4	0.74	-	-	-	-	-	-

Subgroup analysis of N-glycans between sexes revealed significantly increased fucosylation and reduced branching in female plasma, as well as a minor increase in sialylation and branching in the female cerebellum. The cortex trends similarly to the cerebellum, though the differences do not reach statistical significance. For plasma samples male=8, female=6. For brain samples male=6, female=4. *p*-values <0.05 using an unpaired two-tailed t-tests assuming unequal variance performed for sex comparisons of glycan categories shown in bold.
