# Supplementary material for: Mammalian brain glycoproteins exhibit diminished glycan complexity compared to other tissues
Source: Nat Commun. 2022 Jan 12;13:275. doi: 10.1038/s41467-021-27781-9 (PMC8755730; doi:10.1038/s41467-021-27781-9)
Supplement: Supplementary file 3 — Description of Additional Supplementary Files [file 41467_2021_27781_MOESM3_ESM.pdf]

Mammalian brain glycoproteins exhibit diminished glycan complexity compared to other tissues

Sarah E. Williams,[1,2,3] Maxence Noel,[2] Sylvain Lehoux,[2] Murat Cetinbas,[4] Ramnik J. Xavier,[5,6] Ruslan Sadreyev,[4] Edward M. Scolnick,[1,6] Jordan W. Smoller,[1,6,7] Richard D. Cummings,[2,#] and Robert G. Mealer[1,2,6,7,#,*]

Description of Additional Supplementary Files

File Name: Supplementary Data 1
Description: Individual protein glycan abundance across mouse brain regions. Data presented as mean percent abundance. For N-glycans, n=6 for each brain region. For O-glycans, CTX=3, HIP=2, STR=4, CBLM=4.

File Name: Supplementary Data 2
Description: Brain protein N-glycan structure, name, mass, and characteristics.

File Name: Supplementary Data 3
Description: Sex comparison of mouse protein N-glycan abundance in plasma, cortex, and cerebellum. For plasma samples male=8, female=6. For brain samples male=6, female=4. p-values <0.05 using an unpaired two-tailed t-tests assuming unequal variance performed for sex comparisons of individual glycans shown in bold.

File Name: Supplementary Data 4
Description: RNA expression levels of glycosylation genes detected in mouse cortex and cerebellum. EdgeR method was used for differential expression analysis of RNAseq data with gene cutoffs of 2-fold change in expression value and false discovery rates (FDR) below 0.05. *Abbreviations: ID - Ensembl ID; CTX - Cortex; CBLM - Cerebellum; AVG - Average; SEM - Standard Error of the Mean; logFC - log Fold Change; logCPM - log Copies Per Million; FDR - False Discovery Rate.

File Name: Supplementary Data 5
Description: Human glycosylation-related genes included in FUMA analysis.
