# Supplementary material for: Mammalian brain glycoproteins exhibit diminished glycan complexity compared to other tissues
Source: Nat Commun. 2022 Jan 12;13:275. doi: 10.1038/s41467-021-27781-9 (PMC8755730; doi:10.1038/s41467-021-27781-9)
Supplement: Supplementary file 9 — Reporting Summary [file 41467_2021_27781_MOESM9_ESM.pdf]

nature research

Corresponding author(s): Robert G. Mealer

Last updated by author(s): Nov 17, 2021

Reporting Summary

Nature Research wishes to improve the reproducibility of the work that we publish. This form provides structure for consistency and transparency in reporting. For further information on Nature Research policies, see our Editorial Policies and the Editorial Policy Checklist.

Statistics

For all statistical analyses, confirm that the following items are present in the figure legend, table legend, main text, or Methods section.

n/a	Confirmed	
☐	☒	The exact sample size (*n*) for each experimental group/condition, given as a discrete number and unit of measurement
☐	☒	A statement on whether measurements were taken from distinct samples or whether the same sample was measured repeatedly
☐	☒	The statistical test(s) used AND whether they are one- or two-sided<br>*Only common tests should be described solely by name; describe more complex techniques in the Methods section.*
☒	☐	A description of all covariates tested
☐	☒	A description of any assumptions or corrections, such as tests of normality and adjustment for multiple comparisons
☐	☒	A full description of the statistical parameters including central tendency (e.g. means) or other basic estimates (e.g. regression coefficient) AND variation (e.g. standard deviation) or associated estimates of uncertainty (e.g. confidence intervals)
☐	☒	For null hypothesis testing, the test statistic (e.g. *F*, *t*, *r*) with confidence intervals, effect sizes, degrees of freedom and *P* value noted<br>*Give P values as exact values whenever suitable.*
☒	☐	For Bayesian analysis, information on the choice of priors and Markov chain Monte Carlo settings
☐	☒	For hierarchical and complex designs, identification of the appropriate level for tests and full reporting of outcomes
☒	☐	Estimates of effect sizes (e.g. Cohen's *d*, Pearson's *r*), indicating how they were calculated

Our web collection on statistics for biologists contains articles on many of the points above.

Software and code

Policy information about availability of computer code

Data collection	FlexControl v3.4
Data analysis	Microsoft Excel v16.35, GraphPad Prism v8.4.2, FUMA GENE2FUNC Tool v1.3.6 RRID:SCR_017521, FlexAnalysis v3.4, mMass v5.5.0, STAR aligner 20201, HTSEQ v0.11.2, EdgeR v3.28.1, Python v3.7.4, R v4.0.2, LiCOR Image Studio v5.2.5, GlycoWorkbench v2.1

For manuscripts utilizing custom algorithms or software that are central to the research but not yet described in published literature, software must be made available to editors and reviewers. We strongly encourage code deposition in a community repository (e.g. GitHub). See the Nature Research guidelines for submitting code & software for further information.

Data

Policy information about availability of data

All manuscripts must include a data availability statement. This statement should provide the following information, where applicable:

- Accession codes, unique identifiers, or web links for publicly available datasets
- A list of figures that have associated raw data
- A description of any restrictions on data availability

The data generated in this study are included in this published article and its supplementary information files, and Source data are provided with this paper. The raw MS glycomics data generated in this study have been deposited in the GlycoPOST database under accession code GPST000213 [https://glycopost.glycosmos.org/entry/GPST000213] (wild-type and A391T mutant glycomics data). The RNAseq data generated in this study have been deposited in the NCBI's Gene Expression Omnibus under GEO Series accession number GSE184516 [https://www.ncbi.nlm.nih.gov/geo/query/acc.cgi?acc=GSE184516] (wild-type and A391T mutant RNAseq data). Human gene expression data is publicly available from the Genotype-Tissue Expression (GTEx) Portal, Version 8 [https://gtexportal.org]. Protocols for glycomics analysis are publicly available through the National Center for Functional Glycomics [www.ncfg.hms.harvard.edu].

Field-specific reporting

Please select the one below that is the best fit for your research. If you are not sure, read the appropriate sections before making your selection.

- [x] Life sciences
- [ ] Behavioural & social sciences
- [ ] Ecological, evolutionary & environmental sciences

For a reference copy of the document with all sections, see nature.com/documents/nr-reporting-summary-flat.pdf

Life sciences study design

All studies must disclose on these points even when the disclosure is negative.

Sample size	The number of samples analyzed was based on our related study of brain glycosylation in mice, where groups of ~4 wild-type vs mutant mice provided adequate power to detect even subtle differences between genotypes with minimal group variation (see Mealer et al, 2021 , https://www.biorxiv.org/content/10.1101/2020.12.22.424076v1). In additional, consultation with Dr. Sadreyev regarding adequate sample size for RNAseq of clonal littermate mice was consistent with groups of 4.
Data exclusions	No data were excluded from the study
Replication	Each experiment was performed with the number of samples as described above and included in the figure legends. For glycomics, positive control samples of previously purified glycans were included during MALDI-MS for quality control and to adjust for batch effects.
Randomization	Randomization was not utilized in our study as we began with a defined number of animals (~6/group) and performed parallel analyses from the identical samples using multiple techniques. For example, one mouse brain would be separated into cortex and cerebellum during dissection and processed simultaneously with other samples for glycomics and RNA seq. Additional co-variates were not considered in our study design as all mice were litter mates on a clonal genetic background.
Blinding	MS Glycomics data collection was blinded for each sample within each batch from each region (cortex, cerebellum, etc.) using coded numbers. Analysis was completed after all data was obtained and unblinded using the code. RNAseq samples from each region were coded and processed blindly.

Reporting for specific materials, systems and methods

We require information from authors about some types of materials, experimental systems and methods used in many studies. Here, indicate whether each material, system or method listed is relevant to your study. If you are not sure if a list item applies to your research, read the appropriate section before selecting a response.

Materials & experimental systems

n/a	Involved in the study	
[ ]	[x]	Antibodies
[x]	[ ]	Eukaryotic cell lines
[x]	[ ]	Palaeontology and archaeology
[ ]	[x]	Animals and other organisms
[x]	[ ]	Human research participants
[x]	[ ]	Clinical data
[x]	[ ]	Dual use research of concern

Methods

n/a	Involved in the study	
[x]	[ ]	ChIP-seq
[x]	[ ]	Flow cytometry
[x]	[ ]	MRI-based neuroimaging

Antibodies

Antibodies used	Mouse anti-actin antibody was purchased from Abcam (ab8226).<br>Goat anti-Mouse IgG IRDye 680RD was purchased from LiCOR (925-68070).
Validation	Mouse anti-actin antibody validation is described on manufacturers website as "suitable for: ICC/IF, IHC-P, WB, Reacts with: Mouse, Rat, Human and Isotype: IgG1." (https://www.abcam.com/beta-actin-antibody-mabcam-8226-loading-control-ab8226.html).<br><br>Goat anti-Mouse IgG IRDye 680RDMouse antibody validation is described on manufacturers website (https://www.licor.com/bio/reagents/irdye-680rd-goat-anti-mouse-igg-secondary-antibody). "Isolation of specific antibodies was accomplished by affinity chromatography using pooled mouse IgG covalently linked to agarose. Based on ELISA and flow cytometry, this antibody reacts with the heavy and light chains of mouse IgG1, IgG2a, IgG2b, and IgG3, and with the light chains of mouse IgM and IgA. This antibody was tested by dot blot and and/or solid-phase adsorbed for minimal cross-reactivity with human, rabbit, goat, rat, and horse serum proteins, but may cross-react with immunoglobulins from other species. The conjugate has been specifically tested and qualified for Western blot and In-Cell Western™ assay applications."

Animals and other organisms

Policy information about studies involving animals; ARRIVE guidelines recommended for reporting animal research

Laboratory animals	C57BL/6J mice from JAX labs from both sexes were used in this study and were 12 weeks old at the time of tissue harvest. Animals are housing in the MGH Center for Comparative Medicine (CCM) Animal Facility, with 12 hour light/dark cycles, controlled temperature of 68 - 79°F, and relative humidity of 30 - 70%.
Wild animals	This study did not use wild animals
Field-collected samples	This study did not include samples collected in the field.
Ethics oversight	All mice were housed and maintained in accordance with the guidelines established by the Animal Care and Use Committee at Massachusetts General Hospital under protocol #2003N000158.

Note that full information on the approval of the study protocol must also be provided in the manuscript.
